# Reviews and Book Notices

**Published:** 1878-12

**Authors:** 


### Reviews and Book Notices.

THE LAW OF POPULATION—ITS CONSEQUENCES AND ITS BEARING UPON HUMAN CONDUCT AND MORALS. By Annie Besant. Authorized American from the 25th thousand, English Edition.

This is one of the issues of what may be most fittingly termed the *pseudo-philanthropical press.* Commencing with a statement of the Malthusian doctrines concerning the disproportionate ratios of increase of the earth's population and the production of food, it is made clearly apparent that in certain countries the population is becoming too great for the supply of food. This approach to starvation of the masses continually grows worse instead of better. Consequently it is desirable in densely crowded communities to do something to limit the propagation of the species, if we would obviate the tendency to increasing misery.

The author then reviews the evils which grow out of an attempt to limit population by postponement of wedlock till a late period of life. This course leads inevitably to prostitution of great numbers of women, and spreads disease and immorality in every direction. This method, therefore, is pronounced a failure.

So far there is no fault to be found with the essay. It reads like the work of a true philanthropist. But now comes the descent from the sublime to the ridiculous Artificial interference with the results of copulation is announced as the true way of getting around all these frightful difficulties which render the human race a burden to itself. A soft sponge in the vagina of every wedded wife—and no woman hereafter need go unwedded through fear of offspring—is the unfailing panacea which shall keep the balance true between mouths and food! No other method is considered by our author equal to this!! I am not sure but it is a sufficient criticism to say that for the publication of this opinion the authoress was sentenced by a court of law, in that very land which she desired to relieve, to pay a heavy fine and to suffer a limited term of imprisonment. Evidently the morality of such philanthropy is not very highly esteemed.

It is not, however, with the morality of these expedients that we have to do. What shall we say of them when viewed from the stand-point of the physician? Upon this subject, the testimony of gynæcologists is uniform, to the effect that such methods infallibly lead to uterine disease. This should be sufficient to condemn the practice. If we are to resort to art for relief in these matters, let us be consistent and thorough. Let us have some more sanitary laws and some more sanitary officials, to regulate our lives. It is an open question whether the practice of spaying would not be preferable to such nuisances as the advice of this pamphlet would create. When our sexual relations have been thus perfected and re-adjusted to the wants of *civilization*, we shall have a lesson in the results of pin-hole philanthropy at which angels—if not men—will stand aghast.

Man is a fine animal as he stands; but if he is to be improved in any respect we must be content to work with instead of against Nature. I cannot but think that it would be well for these agitators, who so greatly desire to improve upon the relations which exist between the sexes, to imbibe a little of the wholesome spirit displayed by Miss Polly Baker, when she was arraigned before the judges, in the good old times, to answer for the birth of a fifth illegitimate child. She addressed the court (so runs the ancient chronicle) to the following effect:

"She acknowledges the fact; says that twice she paid the fines imposed, and twice was punished for lack of means with which to pay the fines; thinks all this may be in accordance with the law, but argues on the unreasonableness of the law which punishes her, who at the risk of her life, has brought five children into the world, maintained them well by her own industry without burdening the township, and fitted them to become good subjects of the king; that she has wronged no one, unless it be the minister or justice, by reason of their having missed a marriage fee; that she would have much preferred to be married, never having refused an offer of marriage, but on the contrary, accepting the first one she had, and the result was she unhappily lost her own honor while trusting too much to *his*; that she had been most severely punished, while he had been raised to an office of honor and profit, being, in fact, one of the judges in that very court in which she was being tried, but not upon that occasion present. She reminds the court that, according to their belief, she has offended heaven and must suffer eternal fire, and urges if that is not sufficient punishment without their intervention; at the same time declares her unbelief in such doctrines as that Heaven is angry at her having children, when to the little she had done toward it God had been pleased to add his divine skill and admirable workmanship in the formation of their bodies, and crowned it by furnishing them with rational and immortal souls. She further urges that if men must be eternally making laws, they ought at least to leave alone the natural and useful actions of men, and not by their prohibitions to turn such actions into crimes. They ought to punish the bachelors, who do nothing to populate the country, but leave unproduced (which is little better than murder) hundreds of their own posterity, while young women are most severely punished for obeying the first great command of nature and of nature's God, to increase and multiply—a duty from the steady performance of which nothing has been able to deter her; and that the court, instead of punishing her, ought to erect a statue to her memory."

It is also recorded that one of the judges married her the next day, and she had fifteen more children.

Verily, if we must choose between Miss Baker on the one hand and Mrs. Besant with her sponges on the other, give us Miss Baker every time, and the bread and butter question will surely take care of itself. H. M. L.

INSANITY IN ANCIENT AND MODERN LIFE, WITH CHAPTERS ON ITS PREVENTION. By Daniel Hoek Tuke, F. R. C. P., Lond. London: MacMillan & Co. Chicago: Jansen, McClurg & Co., 1878; cloth pp. 226, $1.75.

Dr. Tuke, the author of the present volume, is already favorably known to the profession by his admirable treatise entitled,

"Illustrations of the Influence of the Mind upon the Body," and by his joint authorship with Dr. Bucknill of the best work on Psychological Medicine in the English language. The present, like the former publication, shows earnest, patient and laborious research and a capacity to draw judicious conclusions from materials collected.

The book is divided into three parts.

Part first considers the prevalence of the causes of insanity among the nations of Antiquity. He begins with a discussion of the causes that prevail generally in the production of insanity, enumerating: first, intoxication, including the abuse of stimulants of all kinds, their effect on the individual and on his offspring; second, defective nutrition—including bad sanitary arrangements—which leads to malnutrition and exhaustion of the nervous centers and degeneration of race; third, various causes, chiefly moral, which excite or depress the emotions profoundly, and lastly intellectual strain.

These various ætiological factors are considered in connection with the mental aspects of the prehistoric man; of the ancient Jews and Egyptians—and of the Greeks and Romans. In this discussion our author has expended a vast amount of time in historical research, and his deductions are well worth the careful attention of the profession, as they are of great practical importance to the laity.

In the conclusion of this part of his subject he says: "After the necessarily imperfect sketch we have drawn of the psychological bearings of ancient history, as to the prevalence of the main causes of insanity, we endeavor to draw a general conclusion, we appear to be warranted in saying that mental disease was not likely to be largely developed among the primative races, that the causes of mental disorder must have exerted a very considerable influence upon the four important nations referred to, less in their earlier, much greater in their later and highly organized condition."

"In favor of the nations of antiquity as compared, with England, may be enumerated less dram and beer drinking and fewer half-starved and diseased children reared."

"And, again, the very benevolence and consideration which a humane nation like England displays towards the poor, and those of feeble mind, or who are becoming insane, instead of allowing them to perish, favors alike the accumulation of insane persons, and the propagation of the disease by such, before they are placed in restraint or after their recovery. Feeble mental constitutions perished by the way in Egypt; sons, probably affected with moral insanity, as evinced by disobedience to parents, etc., were stoned to death in Palestine; homicidal men killed and were killed in the wars of Greece and Rome, and defective children were thrown down the Tarpeian rock. There was not, therefore, so much feebleness, moral insanity, or homicidal impulse transmitted to the next generation in the old heathen or Jewish, as compared with modern Christian populations."

The second part of the book is devoted to the consideration of the subject in its relations to modern life, with chapters on: 1st, insanity, chiefly in relation to the working classes; 2d, insanity in relation to the higher classes, and 3d, facts and figures in regard to the increase of insanity. In these chapters will be found an interesting psychological comparison between modern and ancient life and a satisfactory explanation of the greater prevalence of insanity at the present time than formerly.

The third part is a discussion of the important question of prevention. In this he enunciates as a cardinal principle that the brain is the organ of the mind and subject to the laws of physical life in general, and to those of cerebral life in particular—that proper nourishment, assimilation and discharge of effete matter are as necessary for the mind organ as for any of the other viscera of the human body.

Chapter IX. is entitled "Warnings of Danger," and is given to the consideration of certain symptoms of brain lesion that our author regards as of special prominence in the onset of insanity such as insomnia, cephalalgia, mental inaptitude, listlessness, various emotional disturbances and indecision of purpose. He also, in the same chapter, draws an excellent picture of the insane temperament.

In chapters X. and XI. are considered the importance of cheerfulness, mental rest and proper diet in arresting the facile descent to madness.

These chapters are admirably written, full of valuable facts and well worth the careful study of every physician.

The book closes with several pages of tersely written psychological axioms, rich in wholesome counsel, for those unfortunate enough to possess the insane neuroses. D. R. B.

NERVOUS DISEASES: THEIR DESCRIPTION AND TREATMENT. By Allan McLane Hamilton, M. D., one of the attending physicians at the Epileptic and Paralytic Hospital, Fel. N. Y. Acad. Med., etc., etc., 8vo., pp. 512, with 53 Illustrations. Philadelphia: H. C. Lea, 1878.

After having read the various and widely differing reviews which have appeared, we took up Dr. Hamilton's book in a very uncertain mental condition, but with every intention to ascertain the real character of the volume in hand.

A very careful examination has fully demonstrated that the many favorable notices which have appeared in the various journals have been in consonance with the merits of the book, and, further that the four evidently *personal* and self-condemnatory reviews, which stand arrayed against the honest and truthful voice, have emanated from some unworthy rival, from one and the same person. Such scurrilous attacks are not only grossly unfair, but are a reflection upon the profession. Although eventually suicidal, such practices succeed for a while in influencing the unwary.

The author's self-imposed task was no simple one; he has stated in his "preface" that his object has been to produce a concise and practical work, whereby the diseases of the nervous system may be, if possible, more comprehensible to his readers than they have been by former elucidations, and we do not hesitate to affirm that he has produced the best text-book, considered in all its relations, written in the English language. Hammond's excellent work is more bulky without conveying any more information; then, too, it contains some views which have not been accepted by other neurologists, or in any wise satisfactorily demonstrated. Like lectures in general, Charcot's and Wilke's works are very instructive and interesting reading for the general and special practitioner, but are too verbose and too limited in subjects for the use of students. Dr. Hamilton's book is especially adapted for the use of students; it is essentially a text-book. Its arrangement is excellent. Beginning with the cerebrum and its membranes, it follows a course therefrom to the cerebellum, spinal cord, medulla oblongata, cerebro-spinal nerves, and, finally, to the peripheral nerves. The book first introduces us to what and how to observe and record in patients, gives directions for making post-mortem examinations in nervous cases, and very satisfactorily explains the use of the various instruments needed for a proper diagnosis, namely, the thermometer, æsthesiometer, piesmeter, dynamometer, ophthalmoscope, electrical apparatus, rubber muscles, etc. Wood cuts illustrate the text. The diseases of the cerebral meninges are excellently described and diagnosticated, reports of clinical cases materially adding to the elucidation of each subject. The diagnosis of ante-tubercular meningitis (acute hydrocephalus) is a model of excellence. A clearly described article on cerebral hyperæmia brings us to the best chapter in the book, cerebral hæmorrhage. Twenty-nine pages are devoted to this subject, which is treated in a masterly manner. No one can read this article without gaining a better understanding of this affection. We notice an error on page 95, wherein the author's calculation has been a little at fault. We presume Dr. H. refers to the *twenty* cases, with no history as to cause, when speaking of those wherein the attack came on during the *night*, instead of *thirty*, as is stated in the text.

Of cerebral anæmia, embolism, and thrombosis, the student shall find a concise and clear exposition. Stomachic and auditory vertigo are not omitted. Every student who has studied cerebral softening in other works must hail with delight the author's most lucid and full elucidation of this interesting though difficult subject. This chapter alone is worth the price of the book. Aphasia is well described; excellent wood-cuts illustrate certain brain localizations. The chapter on "Brain Tumors," ranks next to that on cerebral hæmorrhage in point of excellence. It is a very complete chapter; and, with the assistance of Petrina's (of Prague) directions for their localization, the practitioner may diagnose these tumors with some degree of certainty. The woood-cuts accompaning the text are very good.

Spinal affections are fully exposed in the light of our latest knowledge. We have only time and space to call attention to the fact that " tetanus " is an able article, its value being greatly enhanced by the figured map of the Long Island district. Dr. H. has embodied his personal inquiries upon this district in the volume. The other exhaustive and eminently practical article in this book is upon epilepsy; a better one could scarcely be written. Every practitioner who thinks the routine use of the bromides is the best and proper treatment of this affection should certainly purchase the author's work, read and digest his remarks upon the treatment of this obscure disease. Bulbar paralysis, cerebro-spinal meningitis, alcoholism, hydrophobia, hysteria and its varieties, chorea, paralysis agitans, and Graves' disease, are all well described. Neuralgia and other diseases of the peripheral nerves are equally well treated of. An important feature for students is an appendix of 95 excellent formulæ. A full index closes this interesting and valuable production.

We have noticed some typographical errors, unavoidable in a first edition. Take the book all in all, considering the author's concise and lucid style, his rare ability in compressing a large amount of information into small space, and the fact that he has elucidated his subject in the light of the latest researches, we assure the student and general practitioner that no better book of its kind exists than " Hamilton on Nervous Diseases."

F. D. B.

A Guide to the Practical Examination of Urine. For the use of Physicians and Students. By James Tyson, M. D. Cloth, pp. 172. Second edition, 1878.

Little need be said to introduce this second edition of Dr. Tyson's work. Special credit is due the author for the minute attention which he has paid to the examination for albumen. We regret, however, that the author has not incorporated the very simple methods of estimating the quantity of albumen approximately by a dilution of the urine. The suggestion, we think, was made by Dr. Roberts, of Manchester.

There is another omission which we also note, namely, the estimation of urea by the diminished specific gravity, after subjecting the urine to the action of the hypochlorite solution. This method is due to Dr. G. B. Fowler.

We should like also to have seen some mention made of the probable relation of the presence of carbonate of ammonia in the blood, to the phenomena usually referred to uremia.

Although we refer to what we consider omissions, the reader will be amply repaid by the accuracy of detail which the author displays on those subjects that he has developed.

R. T.

TRANSACTIONS OF STATE MEDICAL SOCIETIES.

1. *Transactions of the Medical and Chirurgical Faculty of the State of Maryland. Eightieth annual session, held at Baltimore*, April, 1878.

2. *Transactions of the Iowa State Medical Society*, 1877–78. Volume III., pp. 196.

3. *Transactions of the Twenty-fifth annual meeting of the Medical Society of the State of North Carolina, held at Goldsborough*, May 18th, 1878, pp. 103.

4. *Transactions of the Medical Association of the State of Alabama. Thirty-first session*, 1878, pp. 311.

1. The *Faculty of Maryland* knows well to honor its dead. It paid a fitting tribute to the memory of its distinguished member, Professor Nathan R. Smith (who died July 3, 1877), in the address and biographical sketch by S. C. CHEW, M. D., and by adorning the transactions with the picture of the departed friend.

Professor ALAN P. SMITH, reports fifty-two successive cases of lithotomy without a single death. "Of these, 16 were below five years of age, 13 between five and ten years, 11 between ten and twenty years, 5 between twenty and forty years, and 7 between forty and seventy-five, 4 more below two years of age, the youngest being twenty-two months. The oldest was seventy-one years. Of the whole number, only two were negroes; these, curiously, were the youngest, of twenty-two months, and the oldest, seventy-one years." With the exception of six cases, the operation was performed with the lithotome of his father, the late N. R. Smith; and to the use of this instrument, he attributed in a great measure the satisfactory result of the operations.

The calculi removed varied in composition and size; the largest one measured 13 ctm. in circumference, and weighed 6 decagrams. In a few cases after hæmorrhage occurred, in two instances partial non-retention of urine was the result, and in one case there remained a small fistulous opening in the perineum.

The most interesting case of the whole series was a middle-aged man with a double penis. "The organs were separated above by a deep sulcus, below which they were closely united. They were slightly under the average normal size, and were unprovided with any prepuce. The one upon the right of the median line was normal in every respect, being traversed by an urethra beginning at the extremity of the glans, while the one on the left had the urethral opening below, and just in advance of the scrotum. From this point forward to the glans, the organ was perfectly solid. * * * The scrotum was natural in every respect, and contained two testes of normal size. Upon making an examination, I, of course, passed the sound into the urethra of the right hand penis. The instrument slipped readily into the bladder, but I could not detect by its aid the slightest symptom of calculus. Very much surprised at this, I asked the patient through which opening he passed his urine, and was informed that he used both; and, what was more curious still, that he could use either at will, or that he could first pass a quantity of urine through one, and immediately after discharge about the same amount through the other. Then, for the first time, it occurred to me that there were two bladders; and calling for two utensils, I desired him to first discharge from the right hand organ. From this there flowed a quantity of clear, amber-colored, healthy urine; while, when directly afterwards the same act was performed by the one on the left in a separate vessel, the fluid was ammoniacal and turbid with mucus and pus. Then, at once the case was clear; he had two bladders. The one which I had not yet examined contained the stone sought for. I then attempted to pass my sound into the second urethra, but found the canal contracted and tortuous. Substituting a bougie tipped with steel, I was rewarded by striking immediately a large calculus. Several days were spent in dilating and straightening the canal by means of sea-tangle tents. The removal of the stone was readily effected, the incision being made from within, outward with a bistourie caché. Violent hemorrhage ensued after the operation, and continued very profuse in spite of the most strenuous efforts to arrest it, till he became almost perfectly exsanguine. The pulse ceased to beat at the wrist * * and only slight heart sounds could be detected. However, * * the hæmorrhage ceased; he commenced slowly to react, and progressed to perfect recovery."

Dr. THOMAS R. BROWN follows with a consideration of the various modes of treating urethral strictures.

Dr. JOHN S. LYNCH briefly treats of all agents which reduce animal heat, under the names of *apyretics* and *antipyretics*.

DR. P. C. WILLIAMS discusses the much mooted question of *chloroform in obstetrics*. He considers chloroform as safe as any other anæsthetic in obstetrical practice, and advocates its use: 1, "whenever during labor the woman becomes nervous, fretful, and apprehensive;" 2, "when the os uteri remains firm and unyielding, and threatens to produce a long, tedious labor;" 3, when the termination of the labor is delayed by rigidity of the perineum; and, 4, in all obstetrical operations.

DR. I. E. ATKINSON, in his report on materia medica, makes some very sensible remarks in favor of the metric system.

2. Of the many short articles which compose the volume of the *Iowa State Society*, none has interested us so much as the paper on "Headache," by DR. E. H. HAZEN. May be because headache with its hundred-fold variations is a dear old friend of ours. Dr. H. confines his remarks to headache in diseases of the eye and ear, and shows how it is often erroneously regarded as a neuralgia, which has aggravated an existing eye trouble, such as granular conjunctivitis, where it is a symptom indicating the outbreak of inflammation of the cornea; or headache is treated as neuralgia, while the causative disease is entirely overlooked. As instances of this kind, glaucoma, neuro-retinitis and the errors of refraction are mentioned. In regard to headache in neuro-retinitis, we think the writer is in error. If headache is associated with neuro-retinitis, it is not due to the retinal affection, but some intra-cranial trouble is the common cause of both headache and retinits. Br. Carter, in his lectures, very truly says, " Retinal affections, such as neuro-retinitis leading to nerve atrophy, and the like, are even characteristically painless. The attention of the patient is usually first called to them by the discovery that his sight is fading away without pain."

Dr. Wm. Watson made a short report of an " Epidemic of Cerebro-spinal Meningitis," which occurred in Dubuque and vicinity, during the winter of 1871–2. It must be regretted that he had not kept accurate records of his cases to give a more complete account of his observations.

" Pollution of Drinking-water," by Dr. W. D. Middleton, " Puerperal Convulsions," by Dr. C. H. Rawson, and a number of other short papers complete this interesting volume.

3. The very modest volume of the *North Carolina State Society* contains a paper on " Diphtheria," by Dr. Chas. Duffy, Jr., giving a synopsis of the literature, and reporting a number of cases which came under his observation.

Dr. R. H. Lewis follows, with a short report of " Cases of Ophthalmic Practice." He describes a case of corneitis for the sake of illustrating the injurious effect adstringent eye waters have upon the inflamed cornea: and a case of amblyopia from chronic alchoholism, which corroborated the usefulness of the phosphates in such cases.

Dr. W. T. Emmett has selected " The Life and Discoveries of Harvey," as subject for the annual address.

Dr. R. L. Payne's valedictory address is a discourse on " Maternal Impressions," the pith of which seems to be contained in the following utterance (p. 100): " The very fact of the prevalence of a strong belief in ' maternal impressions ' among the women themselves, although not a positive proof of its truth, is, at least, a forcible presumptive evidence in its favor." Sic!

4. By an act of the general assembly in 1875, the *Medical Association of Alabama* was constituted a permanent Board of Health of the State, and the county societies affiliated with the medical association, were made the Boards of Health for their respective counties, under the direction and control of the association. For this reason, sanitary topics figure largely in the transactions of this society. The president, Peter Bryce, made

"Public Hygiene" the subject of his annual message; Dr. GEO. A. KETCHUN spoke of the "Value of Health to the State;" and the Board of Censors, as a committee of public health, gave a lengthy report of the Quarantine Convention which was held at Jacksonville, Florida, in February of this year.

"Hermaphroditism" is the title of a very elaborate dissertation by Dr. JEROME COCHRAN. By the same author is the following paper: "What is Puerperal Fever?" His answer is that puerperal fever is not a disease *sui generis*, but a symptomatic fever attendant upon erysipelatous, pyæmic, or septicæmic affections in puerperal women. This doctrine is now adopted by most writers, but at the time the paper was first read before the Mobile Medical Society in 1875, there was still some diversity of opinions in regard to the nature of puerperal fever.

A lecture on "The Ophthalmoscope and its Uses," by Dr. W. H. SANDERS, and one on "Endometritis," by Dr. W. H. JOHNSTON, conclude the volume.

F. C. H.

A PRACTICAL TREATISE ON THE DISEASES OF THE EAR, INCLUDING THE ANATOMY OF THE ORGAN. By D. B. St. John Roosa, M. A., M. D., etc. *Fourth Edition.* New York: Wm. Wood & Co. Chicago: Jansen, McClurg & Co.

From the brief advertisement of this edition we learn that, with the exception of the Chapter on Diseases of the Internal Ear, "the work remains as in the last impression." In view of this, together with that other fact that the profession is already quite familiar with the former editions of this excellent treatise, we do not think an extended review of the work is called for at this time.

The appearance of a fourth edition of the work in less than five years from the time it was first issued, is certainly sufficient evidence of its popularity.

We regret to find that of a number of errors which appeared in the first edition, several still remain in this. A few mistakes in a new medical work can easily be excused, but to continue them through a fourth edition, if not censurable is certainly inexcusable.

On page 186, in describing the membrana tympani, the author says: "The handle of the malleus divides the membrane into two parts. The anterior part is larger than the posterior." If this be true, we would suggest that the author have the wood cuts on the opposite page as well as the chromo-lithograph, representing the normal membrane changed. Errors which appeared on pages 201 and 217 have been corrected. Page 305, solution of iodine should be solution of iodide of potash. Page 465, the length of the horizontal semi-circular canal is given as being 5 millimeters. While these errors, with others of like character, are not sufficient to seriously impair the value of the work, it does seem that they should have been corrected.

The chapter on diseases of the internal ear has received valuable additions in the way of clinical histories and a more extended discussion of several diseases. In the management of inflammation or hyperæmia of the internal ear, the author very properly suggests that possibly not enough attention has been paid to the protection of the ear from noises. It certainly appears reasonable that rest, a factor so important in the treatment of inflammations of other parts, should not be ignored here.

If there is a doubt as to the cause of the inflammation, the importance of giving the patient the benefit of the doubt by instituting a thorough anti-syphilitic course of treatment is emphasized.

As to electricity, Dr. Roosa still thinks that as an aid in diagnosis it is of no great value, and as a remedial agent he says: "My own experience has been purely negative."

While it is to be hoped that another edition will not appear without a more extended revision, making additions that the progress of the science demands, and wiping out all errors of former editions, we still think that the work has not lost prestige—that it still remains the best practical treatise on diseases of the ear.

W. T. M.

WALSH'S PHYSICIANS' HANDY LEDGER. A Companion to Walsh's Physicians' Combined Call Book and Tablet. By Ralph Walsh, M. D. Chicago: W. T. Keener.

This book is doubtless already known to many of our readers.

It greatly facilitates the work of book-keeping for the physician. It is arranged with lines and columns on the one page for the number of calls, and corresponding lines and columns on the opposite page for the necessary particulars. Those who meditate a change in their system of booking will do well to become acquainted with it. R. T.

